# Dracula’s ménagerie: A multispecies occupancy analysis of lynx, wildcat, and wolf in the Romanian Carpathians

**DOI:** 10.1002/ece3.8921

**Published:** 2022-05-16

**Authors:** Marissa A. Dyck, Ruben Iosif, Barbara Promberger–Fürpass, Viorel D. Popescu

**Affiliations:** ^1^ 61783 Department of Biological Sciences Ohio University Athens Ohio USA; ^2^ Foundation Conservation Carpathia Brașov Romania; ^3^ 61783 Center for Environmental Research University of Bucharest Bucharest Romania

**Keywords:** carnivores, coexistence, human‐dominated, interactions, landscapes, multispecies occupancy

## Abstract

1. The recovery of terrestrial carnivores in Europe is a conservation success story. Initiatives focused on restoring top predators require information on how resident species may interact with the re‐introduced species as their interactions have the potential to alter food webs, yet such data are scarce for Europe.
2. In this study, we assessed patterns of occupancy and interactions between three carnivore species in the Romanian Carpathians. Romania houses one of the few intact carnivore guilds in Europe, making it an ideal system to assess intraguild interactions and serve as a guide for reintroductions elsewhere.
3. We used camera trap data from two seasons in Transylvanian forests to assess occupancy and co‐occurrence of carnivores using multispecies occupancy models.
4. Mean occupancy in the study area was highest for lynx (Ψ_winter_ = 0.76 95% CI: 0.42–0.92; Ψ_autumn_ = 0.71 CI: 0.38–0.84) and wolf (Ψ_winter_ = 0.60 CI: 0.34–0.78; Ψ_autumn_ = 0.81 CI: 0.25–0.95) and lowest for wildcat (Ψ_winter_ = 0.40 CI: 0.19–0.63; Ψ_autumn_ = 0.52 CI: 0.17–0.78)
5. We found that marginal occupancy predictors for carnivores varied between seasons. We also found differences in predictors of co‐occurrence between seasons for both lynx‐wolf and wildcat‐wolf co‐occurrence. For both seasons, we found that conditional occupancy probabilities of all three species were higher when another species was present.
6. Our results indicate that while there are seasonal differences in predictors of occupancy and co‐occurrence of the three species, co‐occurrence in our study area is high.
7. Terrestrial carnivore recovery efforts are ongoing worldwide. Insights into interspecific relations between carnivore species are critical when considering the depauperate communities they are introduced in. Our work showcases that apex carnivore coexistence is possible, but dependent on protection afforded to forest habitats and their prey base.

## Graphical abstract

In this study, we assessed the seasonal distribution and interactions between three carnivore species in Transylvania using multispecies occupancy models. We found that both marginal and co‐occupancy predictors for carnivores varied between seasons. For both seasons, we found that conditional occupancy probabilities of all three species were higher when another species was present indicating carnivore coexistence is high.

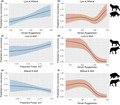

## 1 INTRODUCTION

Terrestrial carnivores are some of the most imperiled species today due to their large home range requirements, high metabolic demands, sensitivity to habitat fragmentation, and persecution by humans (Crooks, 2002; Palomares & Caro, 1999; Ripple et al., 2014; Woodroffe & Ginsberg, 1998). Carnivores can also be important top‐down regulators in ecological communities (Beschta & Ripple, 2009; Ripple & Beschta, 2006; Ripple & Beschta, 2012). The loss of key carnivore species can have devastating ecosystem effects (Effiom et al., 2013; Ripple et al., 2014) and changes in abundance or occurrence of carnivores can trigger trophic cascades (Ripple & Beschta, 2012). As such, the recovery of apex predators as a conservation tool to restore ecosystem functions (termed trophic rewilding) has become increasingly popular (Jørgensen, 2015; Seddon et al., 2014). Trophic rewilding is an ecological restoration strategy used to promote self‐regulating ecosystems (Svenning et al., 2016).

Rewilding efforts in the context of apex predators requires not only an understanding of their ecological interactions within the carnivore guild but also the broader context of these interactions including sources of anthropogenic impacts. Many apex predators readily reestablish in human‐dominated landscapes and exhibit potential coexistence with humans (Chapron et al., 2014; Lamb et al., 2020). Although the effects of apex predator recovery in natural landscapes are relatively well understood, there are significant knowledge gaps regarding the effects of their recovery in shaping species interactions (both intraguild and across trophic levels) in human‐dominated landscapes (Dorresteijn et al., 2015; Kuijper et al., 2016). Interactions between carnivores are complex in nature and are integral to shaping the ecology and structure of wildlife communities. Therefore, examining such interactions in landscapes that harbor viable carnivore populations may provide important insights into the effects of carnivore recovery on the mesocarnivore communities that often dominate landscapes where apex predators have been eliminated.

Grey wolf (*Canis lupus*) and Eurasian lynx (*Lynx lynx*) are top predators in many temperate ecosystems in Europe and Asia, but their co‐occurrence has been severely limited by extirpation of one species (most often wolf). This is particularly the case for most of Western and Central Europe due to a long history of human habitation and persecution of carnivore species. Both wolves and Eurasian lynx are recovering in Europe's landscapes (Chapron et al., 2014; Kaczensky et al., 2013) either through natural range expansion (wolf) or reintroductions and population augmentation (lynx). The European wildcat (*Felis silvestris*) is a mesocarnivore that was once common in Europe and has also been extirpated and currently at the core of reintroduction programs in some European Union states. In this context, the Romanian Carpathians represent one of the few natural areas in Europe that still harbor intact viable populations of all three species and serve as a stronghold for carnivore populations in Europe, despite anthropogenic influences common (hunting, forestry, farming, and livestock production) (Popescu et al., 2016; Salvatori et al., 2002).

While no work has been conducted on understanding the spatial relations and interactions between these three species simultaneously, research exists on pairwise interactions between species, particularly for lynx and wolf. Lynx and wolf are sympatric across most of their range and there is some diet overlap between them. Research addressing coexistence between these species differ in their findings, but recent studies looking at spatial interactions between these species in Europe found that these two apex predators coexist and competition between them is low (Schmidt et al., 2009; Wikenros et al., 2010). In Poland, lynx and wolf territories overlap and researchers concluded that the co‐occurrence of these two species was facilitated by heterogeneous habitat and specialization on different prey (Schmidt et al., 2009). These predictors, habitat heterogeneity and diet, are also explaining competitive interactions between canids and felids in North America, with a lack of interference competition in heterogeneous habitat (Dyck et al., 2022). Therefore, we expect to observe similar co‐existence (high co‐occurrence) and little evidence of interference competition (neutral or positive conditional occupancy values) between lynx and wolf in our study area. Additionally, we expect to observe differences in co‐occurrence based on seasonal changes in these species’ behaviors. For example, the daily movement distances of male lynx are greater during the mating season (January‐March) and for female lynx are greater during periods of extensive kitten care (May‐August) (Jedrzejewski et al., 2002), which could cause increased interactions with wolves as lynx cover a larger geographic area during these periods. Research on wildcats is scarce, but a study conducted in the Jura Mountains of central Europe found no evidence of avoidance between lynx and wildcat (Hercé, 2011). No published research examines interactions between wildcats and wolf. Given the size difference between wolf and wildcats and their different diets, it is likely that the relationship between wildcats and wolf will be similar to that of wildcats and lynx.

In this study, we aimed to address these knowledge gaps by studying the intraguild interactions of two apex carnivores, the Eurasian lynx and the grey wolf, and a mesocarnivore, the wildcat in the Romanian Carpathians using multispecies occupancy models (Rota et al., 2016). Unlike traditional occupancy modeling, multispecies occupancy models allow for the estimation of co‐occurrence probabilities for more than two species and do not assume asymmetric interactions (i.e., dominant and subordinate species). This is useful for estimating co‐occurrence probabilities between species for which there is not a priori knowledge about interspecific relationships or for which there is not an obvious dominant or subordinate species. Multispecies occupancy models also allow for the estimation of marginal occupancy (occupancy of a single species irrelative of other species) and conditional occupancy (occupancy of a single species based on the presence or absence of another species) probabilities in relation to variables of interest (e.g., altitude). This approach has been used effectively to assess habitat use, interspecific interactions of carnivores in a variety of landscapes (Dechner et al., 2018; Lombardi et al., 2020; Van der Weyde et al., 2018). Previous research on lynx–wolf and lynx–wildcat interactions suggests a high capacity for coexistence, low interspecific competition, and little to no intraguild killing. However, this research is limited and there has been no work on wolf‐wildcat dynamics or interactions of lynx, wildcat, and wolf in the same region. Additionally, none of the published literature in Europe has been conducted in an area with a fully intact carnivore guild, whereas the Romanian Carpathians have viable, reproducing populations of many large carnivores and meso‐carnivores that have not been extirpated (see study area). This information is crucial to understanding the effects of apex predators on mesocarnivores and the carnivore guild. By using a multispecies occupancy approach, we can analyze complex intraguild interactions and better understand competition and coexistence patterns. Results can elucidate variables and thresholds important for occurrence and coexistence of elusive species and help inform management or reintroduction efforts. Our specific objectives were as follows: (1) evaluate seasonal predictors for occupancy of each species, (2) characterize the spatial relationships (co‐occurrence) of each species in winter and autumn, and (3) identify predictors that facilitate co‐occurrence. Specifically, we analyzed the effects of potentially dominant apex carnivores on the occupancy and detection of a mesocarnivore to understand potential impacts reintroductions of apex predators may have on smaller carnivores. We also evaluated seasonal changes is marginal and co‐occurrence probabilities to better understand how species persist and interact under different environmental conditions.

## 2 MATERIALS AND METHODS

### 2.1 Study area

**FIGURE 1:**
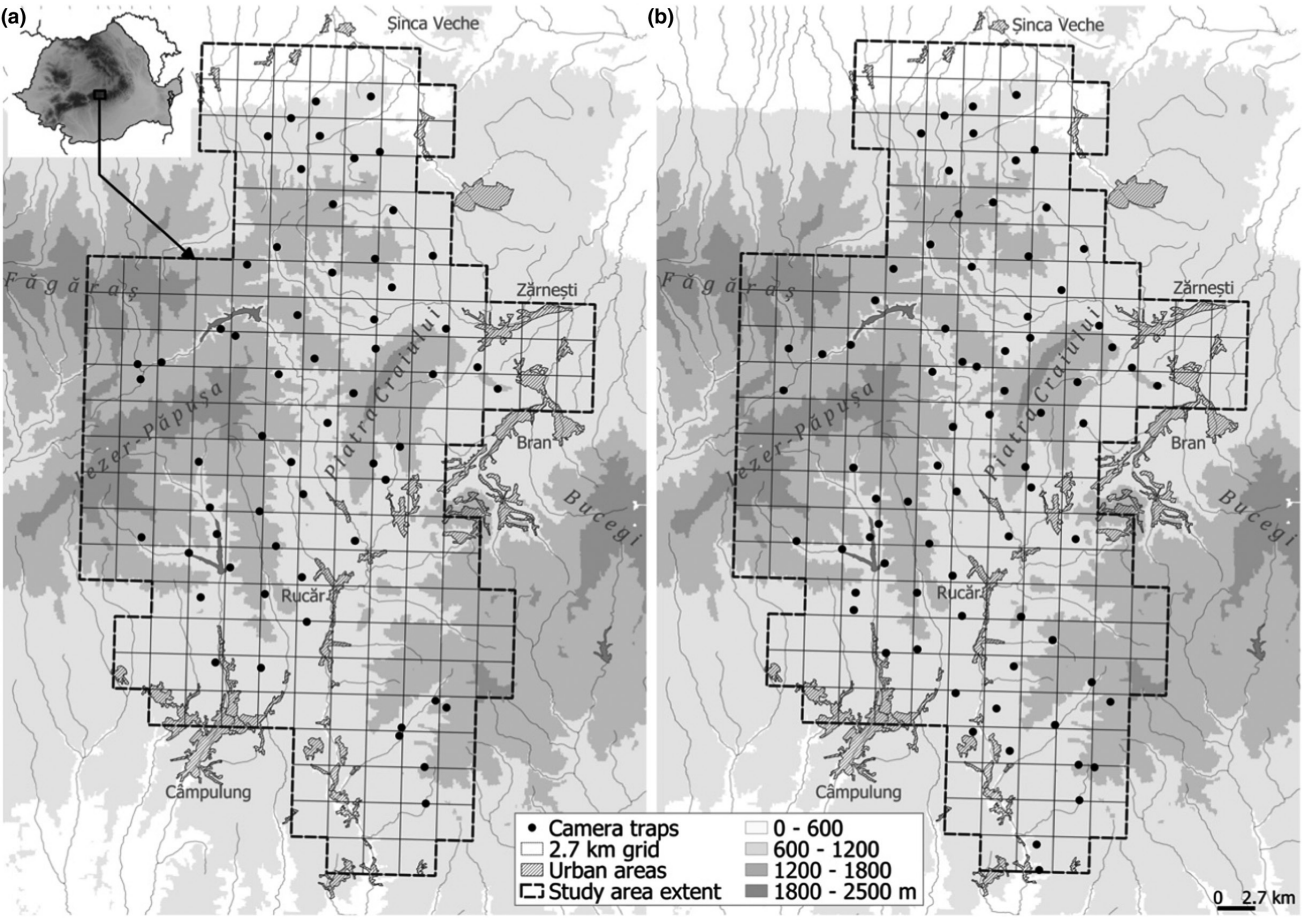
Study area for winter (a) and autumn (b) sessions and the locations of 64 (winter) and 76 (autumn) camera trap stations in Romanian Carpathians, Romania, used for camera trap surveys. Sessions lasted from December 17th, 2018, to March 31st, 2019 (winter), and October 9th, 2019 to January 15th, 2020 (autumn)

The study area is situated in the Southern Carpathians, Romania, covering 1200 km^2^ in the eastern part of the Făgăraș Mountains, Piatra Craiului, and parts of Leaota Mountains (Figure 1). The altitude of the study area ranges from 600 to 2400 m; forests cover most of the area (62%), along with a mosaic of urban–rural landscape and agriculture with significant areas of natural vegetation (22%), and alpine grasslands and subalpine shrubs (16%) (Iosif et al., 2022). Although bisected by a high traffic national road, the area is recognized as a corridor for large carnivore dispersal; the road network is dominated by unpaved forest roads and temporary logging roads (Iosif et al., 2022). The study area harbors an intact mammal assemblage, including large carnivores and meso‐carnivores, brown bear (*Ursus arctos*; density estimate: 17.76 bears/100 km^2^ (95% CI: 15.40–25.74 (Iosif et al., 2021)); lynx (density estimate: 1.6 ± 0.39 *SE* and 1.7 ± 0.38 *SE* lynx/100 km^2^, for winter and autumn respectively (Iosif et al., 2022)), wolf, wildcat, red fox (*Vulpes vulpes)*, European badger (*Meles meles*), and various mustelids; and their prey (roe deer (Capreolus capreolus), red deer (*Cervus elaphus*), chamois (*Rupicapra rupicapra)*, wild boar (*Sus scrofa)*, and leporids, for example, hare (*Lepus europaeus*). Large carnivore hunting is not allowed in the study area. However, anthropogenic disturbance persists in the form of year‐round selective logging, regulated hunting of ungulates, and livestock grazing. The study was part of research conducted by Foundation Conservation Carpathia (FCC) to estimate the density of lynx in the Romanian Carpathians (Iosif et al., 2022).

### 2.2 Camera trapping and environmental variables

We divided the study area into a grid of 2.7 × 2.7 km cells (Figure 1) and removed cells with more than ⅔ of their area exceeding 1800 m altitude and cells more than ½ of their area covered by urban landscape features. From the remaining cells, we sampled every other cell, when it was not possible to reach a selected cell, we used an adjacent cell. Each sampled cell contained a trap station, randomly located within the cell. We conducted two seasons of monitoring: (1) December 17th, 2018, to March 31st, 2019 (winter) and (2) October 9th, 2019, to January 15th, 2020 (autumn). We installed 64 camera trap stations during winter, and 76 during autumn, with high spatial overlap between seasons (Figure 1). Each trap station had two opposite cameras installed at a height of 40 to 60 cm positioned toward animal paths. We used two camera models per trap station, a CuddeBack C1 Model 1279 with white flash for high‐quality color pictures in night conditions, and a Bushnell Trophy infrared camera. Camera traps were installed on animal trails along mountain ridges, mid‐slopes, upper valleys, and bottom of slopes to detect carnivores at various altitudes/habitats. Camera traps were installed 1–2 weeks prior to the start of monitoring to account for additional anthropogenic disturbance from the camera installation process. We checked camera trap stations every two weeks to replace batteries and SD cards.

At each camera trap location, we recorded the presence or absences of anthropogenic disturbance (i.e., logging or settlements) as a binary variable for species detection and occurrence. We also recorded *altitude* (m) via GPS and extracted *distance to stream* (m), *distance to settlement* (m), and *distance to roads* (m) from the camera trap location using Geographic Information Systems (ArcGIS 10.7, ESRI, Redlands CA). Within a 500‐meter buffer around each camera trap location (Lombardi et al., 2020), we calculated the *density of local roads* (km/km^2^), the *proportion of forested area* and a *terrain ruggedness index (TRI)* (Riley et al., 1999). Full covariate descriptions and summaries are available in Table 1.

**TABLE 1:** Names and descriptions of detection and occupancy covariates used in multispecies occupancy modeling for Eurasian lynx (*Lynx lynx*), wildcat (*Felis silvestris*), and grey wolf (*Canis lupus*) in the Romanian Carpathians, 2018–2020

	Name	Description	Type	Summary data
*Detection Covariates*	Distance to stream	Distance from camera to the nearest permanent stream recorded in meters (extracted using GIS)	Numeric variable ranging from 0–1140 m (winter) and 0–1300 m (autumn)	Mean: winter = 237 m autumn = 284 m
	Distance to settlement	Distance from camera to the nearest village recorded in meters (extracted using GIS)	Numeric variable ranging from 224–17,058 m (winter) and 0–17,786 m (autumn)	Mean: winter = 5413 m autumn = 5155 m
	Distance to road	Distance from camera to the nearest paved road recorded in meters (extracted using GIS)	Numeric variable ranging from 0–1302 (winter) and 0–3059 m (autumn)	Mean: winter = 974 m autumn = 1077 m
	Impact	Anthropogenic impact in the immediate vicinity of the camera (recorded by personnel in the field)	Binary variable where 0 = no visible disturbance and 1 = isolated buildings, logging, or villages	winter: 0 = 55, 1 = 9 autumn: 0 = 63, 1 = 13
	Position	Camera position on the landscape (recorded by personnel in the field)	Categorical variable with four levels: ridge, mid‐slope, bottom, valley	winter: ridge (23), midslope (24), bottom (5), valley (12) autumn: ridge (29), midslope (29), bottom (10), valley (8)
	Aspect	Exposure of camera trap location (recorded by personnel in the field)	Categorical variable with four levels: north, south, east, west	winter: north (15), south (25), east (16), west (8) autumn: north (21), south (19), east (20), west (16)
*Occupancy Covariates*	Local road density	Density of roads (km/km^2^) at the grid cell level	Numeric variable ranging from 0.21–0.34 km/km^2^ (winter) and 0.22–0.34 (autumn)	Mean: winter = 0.27 autumn = 0.27
	Terrain Ruggedness Index (TRI)	TRI calculated in R via package “*spatialEco*” using a digital elevation model with resolution 80 × 80 m and two moving window sizes: 5 cells (covering an area of 0.16 km^2^)	Numeric variable ranging from 84–494 (winter) and (autumn); with recommended classification ranges 81–116 ‐ nearly level surface. 117–161 ‐ slightly rugged surface. 162–239 ‐ intermediately rugged surface. 240–497 ‐ moderately rugged surface.	Mean: winter = 223.5 autumn = 217.8
	Proportion forest	Proportion of forest at the grid cell level (extracted from Corine Land Cover 2016 dataset [100 × 100 m resolution] using GIS)	Numeric variable ranging from 0.1–1.0 for both winter and autumn	Mean: winter = 0.78 autumn = 0.75
	Altitude	Altitude of the camera location recorded in meters using GPS by field personnel	Numeric variable ranging from 663–1600 m (winter) and 788–1617 m (autumn)	Mean: winter = 1153 m autumn = 1182 m

### 2.3 Occupancy modeling

We implemented a multispecies occupancy model of two or more interacting species (Rota et al., 2016) in program R 3.5.1 (R Core Team, 2021) via package *unmarked (*Fiske & Chandler, 2011 *)* to explore how environmental and anthropogenic variables affect the marginal occupancy (occupancy without accounting for interactions with other species), co‐occurrence (overlap in marginal occupancy between species), and conditional occupancy (effects of each species presence on other species detection and occupancy) of lynx, wildcat, and wolf in the Romanian Carpathians. Unlike traditional co‐occurrence models, multispecies occupancy models do not require *a priori* assumptions of asymmetric interactions; therefore, species were not considered dominant or subordinate to one another (Rota et al., 2016). Data from the two seasons were analyzed separately, and sessions were divided into 14‐day sampling occasions, with the winter and autumn seasons having eight and seven sampling occasions respectively. Camera trap photos were cataloged by FCC staff and volunteers, and the date, time, location, and species identification were recorded for each animal detection (Iosif et al., 2022). Covariates were checked for correlation using Pearson's correlation tests and Pearson's chi‐squared test (for numerical and factors respectively), those with high correlations *r* > .7 were not included in the same models for the same parameter. We first explored combinations of five detection covariates for species‐specific detection probabilities (Table 1) by comparing models with the same marginal occupancy parameterization for each species. Detection covariates were kept the same for all three species as we did not have a biological reason to vary them between species. We also included the latent presence/absence of every other species as species‐specific detection covariates (e.g., lynx detection predicted by the presence/absence of wildcat and wolf). Although multispecies occupancy models do not assume asymmetric interactions between species, we wanted to explore the possibility that dominant species could exist in our system and affect the presence of other species. Therefore, we also included species‐specific detections of lynx as a function of the latent presence/absence of potentially dominant wolf, and wildcat as a function of lynx and wolf.

From these models, we determined a best model for each season based on Akaike information criterion (AIC), using R package *MuMIn* (Bartoń, 2020). We included the top detection covariates in the models exploring marginal occupancy and co‐occurrence. We then ran a series of models to assess the marginal occupancy of our three species using environmental and anthropogenic variables (Table 1) that were determined *a priori* and we hypothesized it would affect the marginal occupancy of each species. The candidate set of marginal occupancy models was similar for both seasons, models were only removed if variation in covariates was not great enough to allow estimation (i.e., models produced NAs or unreasonable estimates and standard errors). We compared the marginal occupancy models for each season using AIC to identify the best covariates explaining occupancy of each individual species. Using the top covariates from the marginal occupancy analysis, we ran a series of additional candidate models that reflected *a priori* hypotheses regarding pairwise co‐occurrence between lynx and wildcat, lynx and wolf, and wildcat and wolf, and compared the models using AIC and biological relevance (Table S1). Due to data limitations (small sample size), we did not implement a three‐species co‐occurrence parameterization.

## 3 RESULTS

Camera trapping yielded 435 occurrences of all three species in winter and 353 occurrences in autumn, with 6459 and 7083 trap nights for winter and autumn, respectively. We obtained a total of 195 and 179 occurrences of lynx, 69 and 66 occurrences of wildcat, and 171 and 108 occurrences of wolf for the winter and autumn seasons, respectively.

### 3.1 Marginal occupancy

**FIGURE 2:**
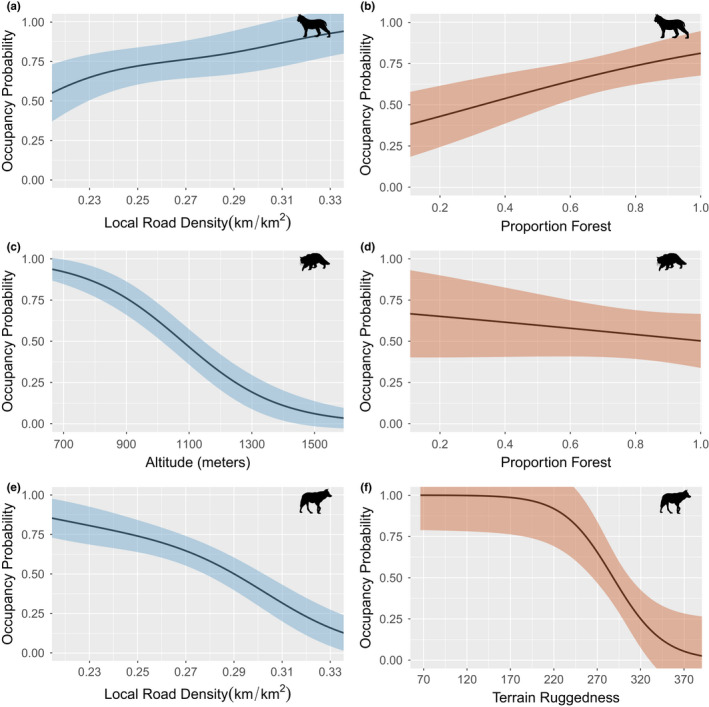
Marginal occupancy probabilities for Eurasian lynx (*Lynx lynx*), European wildcat (*Felis silvestris*), and grey wolf (*Canis lupus*) predicted by the top model for each season and plotted as a function of the marginal occupancy covariates for each species. All variables not included in the plot are assumed fixed at their observed mean. Ribbons represent ±1 SE; blue represents the winter season and red represents the autumn season

Mean occupancy for both seasons was highest for lynx (winter [Ψ = 0.76 95% CI: 0.42–0.92], autumn [Ψ = 0.71 CI: 0.38–0.84]) and wolf (winter [Ψ = 0.60 CI: 0.34–0.78], autumn [Ψ = 0.81 CI: 0.25–0.95]) and lowest for wildcat (winter [Ψ = 0.40 CI: 0.19–0.63], autumn [Ψ = 0.52 CI: 0.17–0.78]) (Figure S1). We found that both marginal and co‐occurrence predictors for lynx, wildcat, and wolf varied between seasons. In winter, local road density was positively associated with marginal occupancy of lynx (Figure 2a) and negatively associated with marginal occupancy of wolf (Figure 2e), while wildcats occupancy decreased with increased altitude (Figure 2c). However, in autumn, marginal occupancy of wolf decreased with terrain ruggedness (Figure 2f), and lynx occupancy increased with forest cover (Figure 2b) while wildcat occupancy decreased with forest cover (Figure 2d).

### 3.2 Co‐occurrence

**FIGURE 3:**
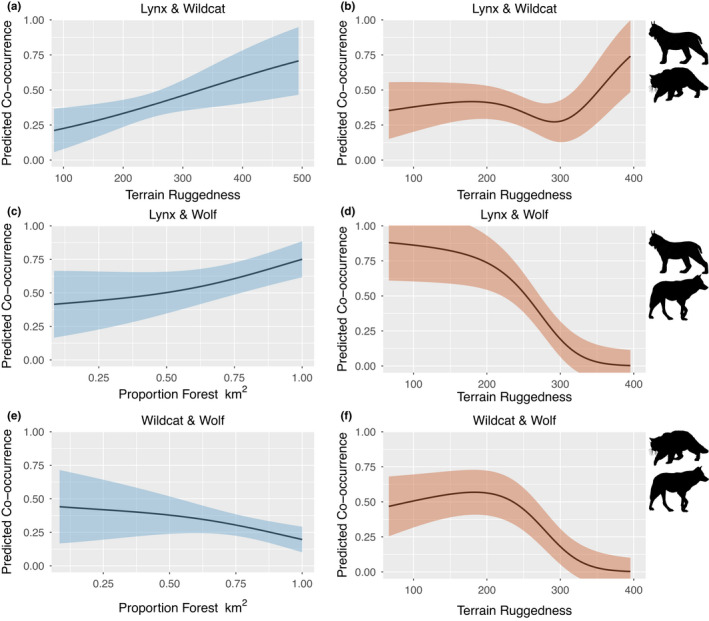
Co‐occurrence probabilities for all pairwise combinations of Eurasian lynx (*Lynx lynx*), European wildcat (*Felis silvestris*), and grey wolf (*Canis lupus*) predicted by the top model for each season and plotted as a function of the co‐occurrence occupancy covariates for each species combination. All variables not included in the plot are assumed fixed at their observed mean. Ribbons represent ±1 SE; blue represents the winter season and red represents the autumn season

We also found differences in predictors of co‐occurrence between seasons for both lynx‐wolf and wildcat‐wolf co‐occupancies. In winter, lynx‐wolf and wildcat‐wolf co‐occurrence were predicted by forest cover (Figure 3b,c), but in autumn, co‐occurrence for both pairs were predicted by terrain ruggedness (Figure 3e,f). Lynx‐wildcat co‐occurrence was predicted by terrain ruggedness for both winter and autumn seasons and was positively associated with terrain ruggedness in both winter and autumn (Figure 3a,d), but in autumn, the relationship was less linear (Figure 3d). In contrast, both lynx‐wolf and wildcat‐wolf co‐occurrence were negatively associated with terrain ruggedness in autumn (Figure 3e,f). In winter, wildcat‐wolf co‐occurrence was negatively associated with forest cover while lynx‐wolf co‐occurrence was positively associated with forest cover, but only at >75% forest cover (Figure 3e,f).

### 3.3 Conditional occupancy

**FIGURE 4:**
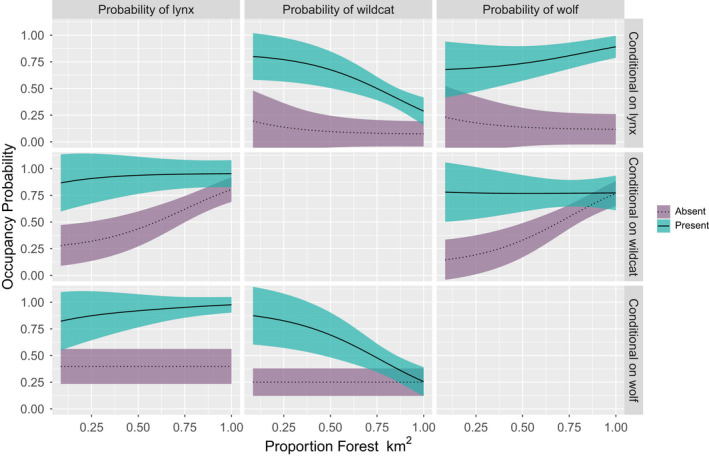
Occupancy probability of lynx, wildcat, and wolf for the winter session, conditional on the presence or absence of each of the other species and proportion of forest in surrounding 9 km. The occupancy probability of the species in each column is conditional on the presence or absence of the species in each row. Lines represent the mean and ribbons represent ±1 SE. All variables not included in the plot are assumed fixed at their observed mean

**FIGURE 5:**
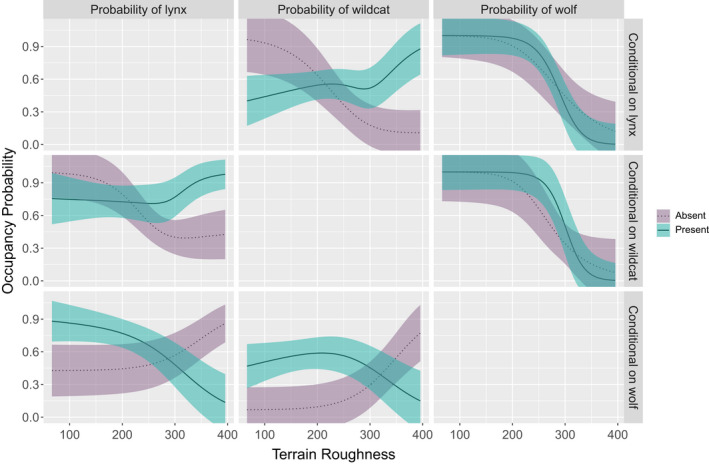
Occupancy probability of lynx, wildcat, and wolf for the autumn session, conditional on the presence or absence of each of the other species and terrain ruggedness

In the winter season, we found that occupancy probabilities of all three species were higher when another species was present, regardless of the species (Figure 4). However, the occupancy probability of wildcat, decreased with increasing forest cover when either lynx or wolf were present (Figure 4), potentially a signal for mesopredator exclusion by apex predators in area of higher suitability. Similarly, in autumn, all species tended to co‐occur, but this relationship was dependent on terrain ruggedness. Occupancy probabilities for both felids, lynx and wildcat, increased with terrain ruggedness when the other felid species was present and decreased when the other species was absent (Figure 5). We observed the inverse relationship for both felids when considering the presence/absences of wolf, such that occupancy probabilities for lynx and wildcat decreased with increased terrain ruggedness when wolf were present and showed a positive relationship with terrain ruggedness when wolf were absent (Figure 5). The presence of lynx and wildcat appeared to have no effect on wolf occupancy.

### 3.4 Detection probabilities

For both seasons, the models that included that latent presence/absence of a potentially dominant species as a detection covariate performed significantly better than those that did not (∆AIC > 5). The top models for each season did not vary in their detection covariates; both models included distance to stream and the latent presence/absence of all species as species‐specific detection covariates. For both seasons, lynx, wildcat, and wolf detections were positively associated with the presence of the other two species (Table S1).

## 4 DISCUSSION

The results from our multispecies occupancy model of lynx, wildcat, and wolves in the Romanian Carpathians indicate that while there are seasonal differences in predictors of occupancy and co‐occurrence of the three species, co‐occurrence of the three species in our study area is high during both seasons. We identified useful predictors of marginal occupancy for each species; in winter were local road density (lynx and wolf) and altitude (wildcat). While in autumn, the best predictors of marginal occupancy were, forest cover (lynx and wildcat) and terrain ruggedness (wolf). We found that co‐occurrence was influenced by environmental variables, forest cover, and terrain roughness, for both winter and autumn. Overall in this heavily forested landscape results from our study indicate that these species coexist but shift patterns of habitat use and co‐occurrence seasonally.

### 4.1 Determinants of occupancy

In winter, local road density was the most important predictor of occupancy for wolf, with higher road density associated with a lower probability of wolf occupancy (Figure 2e). Higher local road density in our study area is associated with higher human disturbance (e.g., limited logging) and habitat fragmentation; this corroborates findings from Jedrzejewski et al. (2004) in northern Poland where wolf had higher occupancy in less disturbed or less fragmented forests. In our study area, the proportion of forest was not an important predictor of wolf occupancy in either season, even though multiple studies have found it to be an important habitat characteristic for wolf (Jedrzejewski et al., 2004; Zlatanova & Popova, 2013) This may be due to the characteristics of our study area which is heavily forested (mean proportion forest =0.78 and 0.75 for winter and autumn monitoring sessions, respectively); thus, forest cover is not a limitation to wolf occurrence. In autumn, terrain ruggedness was the most important predictor of wolf occupancy; when terrain ruggedness index was >200 (moderately to highly rugged areas) the probability of wolf occupancy declined steeply (Figure 2f). This can be explained by the fact that wolf's main prey source in Romania, wild boar (Sin et al., 2019), was documented to prefer less fragmented areas with large beech forest stands in autumn and early winter (Fonseca, 2008). Additionally, red and roe deer, which are also important prey for wolves, are known to move after the rut season (November–December) to more marginal, less topographically‐fragmented areas that provide connectivity to the lower winter grounds (Zweifel‐Schielly et al., 2009). Proportion of forest was a positive predictor of lynx occupancy in autumn, which corroborates other studies that found that lynx occurrence in the Carpathians decreased at low levels of forest cover (Rozylowicz et al., 2010). Local road density was also an important predictor of lynx occupancy in winter, with lynx occupancy positively associated with road density (Figure 2a). While not heavily documented within the *Lynx* genus, other felid species are known to use roads as travel corridors and for hunting and movement within their home range (Bailey, 1993; Bragin, 1986; Gordon & Stewart, 2007; Kerley et al., 2002; Matyushkin, 1977; Rabinowitz et al., 1987). Our results suggest that, in winter, Eurasian lynx are more likely to occupy areas with higher densities of local logging roads; these roads, which in our area are mostly unpaved, dirt roads, may provide easier access to resources within lynx home ranges due to decreased complexity of terrain and decreased snow depth/harder snowpack from vehicle travel. We did not observe this relationship with wildcat, however. Rather, there was a negative relationship between density of local roads and wildcat occupancy in autumn (Figure 2d), which could be an artifact of body size; most documented examples of felids utilizing roads for movement within their home ranges was with larger bodied species (>11 kg). We also did not observe this relationship in winter; however, this is likely an outcome of the importance of altitude for wildcat occupancy, which has a negative relationship (Figure 2c). Higher altitudes are associated with greater snow depth, and while lynx are well adapted to move in deep snow and altitude was not important for lynx occupancy, wildcats have physical limitations that make travel through deep snow more difficult. A study in Switzerland had similar findings whereby wildcats moved to areas free of snow in winter and spring and moved back to high elevations in summer (Mermod & Liberek, 2002). Similarly, in North America, the relationship between Canadian lynx (*Lynx canadensis*) and bobcat (*Lynx rufus*) is mediated by snowpack, with the distribution of the less snow‐adapted, the bobcat, being limited by snow depth at the northern edge of its range (Morin et al., 2020; Reed et al., 2017). Our results for marginal occupancy of lynx, wildcats, and wolf provide insights into both habitat selection and spatial relations for these elusive carnivores in Romania. Our results suggest lynx may use roads for movement, a practice common for other felids of similar body size, but not described in this species. Additionally, we provide further support for previous findings on habitat selection and occupancy for these three European terrestrial predators.

### 4.2 Determinants of co‐occurrence

In winter and autumn, co‐occurrence for lynx and wolf was high indicating that both species have similar habitat requirements. In winter, we found an effect of forest cover on the co‐occurrence of lynx and wolf; co‐occurrence increased with proportion of forest cover >0.75. Increased forest cover is associated with lower productivity of vegetation utilized by roe deer (Melis et al., 2009), the primary shared prey of lynx and wolf. Roe deer abundance is also lower in areas with high forest cover (Melis et al., 2009). Higher lynx‐wolf co‐occurrence in areas expected to have lower roe deer abundance indicates that lynx and wolf are likely partitioning prey resources which would reduce competition. In our study area, wolf also prey on wild boar and red deer (Sin et al., 2019). In autumn, terrain ruggedness was a negative predictor of co‐occurrence for lynx and wolf, such that predicted co‐occurrence was ~0 for the highest values of terrain ruggedness. This relationship is driven by the negative relationship between marginal occupancy for wolf and terrain ruggedness, which is related to prey movements and availability as explained above (Fonseca, 2008; Sin et al., 2019) (Figure 2c). Because marginal occupancy for wolf is ~0 at high terrain ruggedness, co‐occurrence for lynx and wolf is low as well. Additionally, co‐occurrence between wolf and wildcat decreased with terrain ruggedness in autumn (Figure 3f) due to the low marginal occupancy for wolf at high terrain ruggedness. In winter however, co‐occurrence of wolf and wildcat was predicted by proportion of forest such that increasing forest cover resulted in lower co‐occurrence (Figure 3c). In both seasons, the co‐occurrence of lynx and wildcat increased with terrain ruggedness, but the relationship was stronger in winter (Figure 3a,d). This relationship also provides further evidence that the negative relationship observed for lynx and wolf co‐occurrence and terrain ruggedness was driven by wolf marginal occupancy.

### 4.3 Management and conservation implications

The positive effect of wolf and lynx presences on detection of one another, high levels of co‐occurrence in winter, and high levels of conditional occupancy in both seasons (higher occupancy probability when other species is present), for lynx and wolf provide little evidence of interference competition between these apex predators. This suggest that carnivore species may aggregate in certain habitats during winter, potentially driven by prey availability. This corroborates findings from other studies assessing interactions between co‐occurring felids and canids that overlap in resource use. For example, Wikenros et al. (2010) assessed the effects of a recolonizing wolf population on resident lynx in Sweden and found that lynx demographics were unaffected by the presence of wolf. A greater body of literature focuses on the interactions between two similar species, the sympatric bobcat (*Lynx rufus*) and coyote (*Canis latrans*), in North America. A review of literature on this topic reveals a similar story to that of lynx and wolf in the Carpathians, whereby bobcats and coyotes coexist and exhibit little interference competition in most of their range likely due to specialization on different prey and mediation via use of heterogenous habitats (Dyck et al., 2022). Efforts to reintroduce or augment lynx populations also exist in Europe (e.g., Slovenia, Croatia; https://www.lifelynx.eu/). In this context, resident wolf populations should not affect the introduction efforts given that prey base can support both species, and releases occur in highly forested but less topographically fragmented areas. Additionally, our findings also suggest that apex predators have little negative effects on the mesocarnivore, wildcat. This information is useful for management given that wolves are recolonizing their former range in Europe (Chapron et al., 2014). Our findings suggest that wolf would not have negative impacts on wildcat given enough suitable habitat is available. In summary, studying intraguild interactions in an intact system has enabled us to observe and quantify intraspecific interactions among carnivores where they have co‐existed and co‐evolved for centuries. This provides insight into their potential long‐term dynamics for areas where they are recovering naturally or recovering through rewilding efforts. While our study did not include the summer season, our results from two separate and partially overlapping autumn and winter seasons suggest that competition between lynx, wildcat, and wolf is low. However, additional information on the richness and abundance of the prey base, and the spatial and temporal relations between predators and their prey can augment these findings and provide additional management insights in the context of rewilding.

## AUTHOR CONTRIBUTION

**Marissa A Dyck:** Conceptualization (lead); Formal analysis (lead); Investigation (equal); Writing – original draft (lead). **Ruben Iosif:** Conceptualization (equal); Data curation (lead); Methodology (equal); Project administration (equal); Writing – review & editing (equal). **Barbara Promberger–Fürpass:** Conceptualization (equal); Data curation (equal); Funding acquisition (equal); Methodology (equal); Writing – review & editing (supporting). **Viorel Popescu:** Conceptualization (lead); Formal analysis (equal); Funding acquisition (lead); Investigation (equal); Methodology (equal); Project administration (equal); Writing – original draft (supporting); Writing – review & editing (equal).

## CONFLICT OF INTEREST

Authors have no conflict of interest to declare.

## Supporting information

Supplementary Material
