# Supplementary material for: Dracula’s ménagerie: A multispecies occupancy analysis of lynx, wildcat, and wolf in the Romanian Carpathians
Source: Ecol Evol. 2022 May 16;12(5):e8921. doi: 10.1002/ece3.8921 (PMC9109232; doi:10.1002/ece3.8921)
Supplement: Supplementary file 1 — Supplementary Material [file ECE3-12-e8921-s001.docx]

Table S1. Estimates, standard errors (SE), test statistics (Z), and p-values (p) for detection covariates in top multispecies occupancy models for Eurasian lynx (*Lynx lynx*), European wildcat (*Felis silvestris*), and grey wolf (*Canis lupus*) in winter and autumn sessions in the Romanian Carpathians, Romania, 2018-202.

| ***Species/Season*** | **Covariate** | **Estimate** | **SE** | **z** | **p** |
| --- | --- | --- | --- | --- | --- |
| *Lynx/Winter* | Wolf presence | 1.188 | 0.292 | 4.07 | >0.001 |
|  | Wildcat presence | 0.526 | 0.364 | 1.45 | 0.148 |
|  | Distance to stream | 0.207 | 0.125 | 1.65 | 0.099 |
| *Wildcat/Winter* | Lynx presence | 0.432 | 0.385 | 1.12 | 0.262 |
|  | Wolf presence | 0.095 | 0.450 | 0.21 | 0.833 |
|  | Distance to stream | -0.215 | 0.214 | -1.00 | 0.315 |
| *Wolf/Winter* | Lynx presence | 1.164 | 0.304 | 3.83 | >0.001 |
|  | Wildcat presence | 0.305 | 0.461 | 0.66 | 0.509 |
|  | Distance to stream | -0.549 | 0.174 | -3.15 | 0.001 |
| *Lynx/Autumn* | Wolf presence | 0.614 | 0.305 | 2.00 | 0.044 |
|  | Wildcat presence | 0.745 | 0.403 | 1.85 | 0.065 |
|  | Distance to stream | 0.010 | 0.135 | 0.08 | 0.938 |
| *Wildcat/Autumn* | Lynx presence | 0.907 | 0.425 | 2.13 | 0.033 |
|  | Wolf presence | 0.511 | 0.580 | 0.88 | 0.379 |
|  | Distance to stream | -1.214 | 0.352 | -3.45 | >0.001 |
| *Wolf/Autumn* | Lynx presence | 0.728 | 0.307 | 2.38 | 0.017 |
|  | Wildcat presence | 0.045 | 0.503 | 0.09 | 0.928 |
|  | Distance to stream | 0.041 | 0.137 | 0.29 | 0.768 |

Figure S1. Predicted mean occupancy probabilities (with 95% confidence intervals) for Eurasian lynx (*Lynx lynx*), European wildcat (*Felis silvestris*), and grey wolf (*Canis lupus*) for winter (blue) and autumn (red) sessions in the Romanian Carpathians, Romania, 2018-2020.
